# Prediction of prognosis, immunogenicity and efficacy of immunotherapy based on cholesterol metabolism in gastric cancer

**DOI:** 10.3389/fonc.2024.1518010

**Published:** 2024-12-24

**Authors:** Chengjun Zhu, Mengpei Yan, Zhijun Zhang, Yikai Shen, Wangwen Wang, Zetian Chen, Changsheng Cai, Hongda Liu, Zekuan Xu, Zheng Li

**Affiliations:** ^1^ Department of General Surgery, The First Affiliated Hospital of Nanjing Medical University, Nanjing, China; ^2^ Gastric Cancer Center, The First Affiliated Hospital of Nanjing Medical University, Nanjing, China; ^3^ Department of Geriatric Gastroenterology, the First Affiliated Hospital of Nanjing Medical University, Nanjing, China; ^4^ Collaborative Innovation Center for Cancer Personalized Medicine, Nanjing Medical University, Nanjing, China; ^5^ The Institute of Gastric Cancer, Nanjing Medical University, Nanjing, China

**Keywords:** gastric cancer, cholesterol, prognosis, tumor microenvironment, immunotherapy

## Abstract

**Background:**

Cholesterol metabolism plays a crucial role in tumor progression and immune response modulation. However, the precise connection between cholesterol metabolism-related genes (CMRGs) and their implications for clinical prognosis, the tumor microenvironment (TME), and the outcomes of immunotherapy in gastric cancer remains to be fully elucidated.

**Methods:**

Transcriptome data and related clinical information from 675 gastric cancer patients were downloaded from The Cancer Genome Atlas (TCGA) and Gene Expression Omnibus (GEO) databases. A total of 50 cholesterol metabolism-related genes (CMRGs) were identified from the Kyoto Encyclopedia of Genes and Genomes (KEGG, hsa04979). Consensus clustering analysis was used to classify patients into distinct molecular subgroups, while principal component analysis (PCA) was applied to develop a prognostic scoring system for predicting survival and immunotherapy response. The scoring system was validated using three independent cohorts of gastric cancer patients.

**Results:**

Based on 49 CMRGs, 675 gastric cancer patients were categorized into three distinct subgroups with varying prognoses, tumor microenvironment features, and clinical characteristics. Further differential gene analysis and consensus clustering identified two additional subgroups. The prognostic scoring system developed through PCA demonstrated that the high-score subgroup had significantly improved survival, higher tumor mutational burden (TMB), and microsatellite instability (MSI), as well as a greater number of mutated genes, indicating greater sensitivity to immunotherapy. This system was validated in a real-world cohort undergoing immunotherapy. Additionally, the correlation between GPC3 expression and cholesterol levels was confirmed, highlighting GPC3’s potential biological role.

**Conclusion:**

This study highlights the importance of CMRGs in gastric cancer, deepens our understanding of the tumor immune microenvironment, and guides individualized immunotherapy.

## Introduction

Gastric cancer(GC)is the third leading cause of cancer-related deaths worldwide (1). Despite advancements in treatment strategies, the five-year survival rate for GC patients remains low (2, 3). In recent years, immunotherapy particularly checkpoint inhibitor-based approaches has shown promising potential in clinical studies (4). However, tumor heterogeneity continues to pose significant challenges, leading to drug resistance and relapse, which limit the efficacy of immunotherapy. Consequently, a comprehensive understanding of the mechanisms underlying differential responses to immunotherapy, along with the development of reliable tools for predicting prognosis and therapeutic outcomes, is crucial.

The progression of GC is a complex process heavily influenced by interactions between cancer cells and the tumor microenvironment (TME) (5, 6). Among abnormal TME metabolites, cholesterol plays a pivotal role in GC (7, 8).Reprogrammed cholesterol metabolism, a hallmark of cancer cells, promotes their proliferation, survival, and progression by disrupting homeostasis and altering metabolic pathways (9). Cellular cholesterol metabolism is mainly regulated by two key transcription factors, SREBP2 and LXR. SREBP2 transcribes genes that regulate cellular cholesterol levels above cholesterol biosynthesis and uptake, while LXR transcribes genes that regulate cholesterol efflux and inhibit cholesterol uptake to down-regulate cellular cholesterol levels (10). Gastric cancer cells, as rapidly proliferating cells, require high levels of cholesterol to meet functional requirements such as membrane biogenesis and are usually engage active SREBP2 signaling to upregulate cholesterol biosynthesis and uptake. For instance, the cholesterol-derived oncometabolite 25-HC, which is enriched in patients with GC, promotes GC cell invasion by up-regulating TLR2/NF-κB mediated MMP expression (11). Multiple lines of evidence indicate that alterations in the cholesterol pathway can modulate the immune system through diverse mechanisms, eliciting a broad range of responses. Ma et al. revealed that cholesterol accumulation induces CD8+ T cell exhaustion by suppressing IFN-γ and GZMB production, resulting in a non-inflamed tumor immune microenvironment (TIME) and impaired antitumor immunity (12). They proposed cholesterol reduction as a potential strategy to restore T cell function and enhance T cell-based immunotherapy. Ning et al. demonstrated that the combination of simvastatin and PD-1 blockade synergistically enhances antitumor efficacy in GC (13).Currently, the majority of studies tend to concentrate on one or two genes within cholesterol metabolism while tumor development typically arises from the intricate interplay of numerous genes. Hence, it is imperative to comprehensively analyze the interrelationships among multiple genes involved in cholesterol metabolism to discover immune phenotypes and effective treatment strategies.

In this study, we conducted a comprehensive analysis of 675 gastric cancer samples from TCGA and GEO databases to investigate the role of cholesterol metabolism-related genes (CMRGs) in gastric cancer. By examining the expression levels and mutation characteristics of 49 CMRGs, we identified three distinct cholesterol subgroups with unique prognostic and tumor microenvironment profiles. Differentially expressed genes (DEGs) among these subgroups were further analyzed, leading to the identification of two additional gene-based subgroups, characterized by differences in transcriptional expression, somatic mutations, copy number variations, and gene methylation. We developed an innovative prognostic scoring system, Chole-Score, which accurately predicts patient prognosis, immune infiltration characteristics, immune checkpoint inhibitor (ICI) response rates, and antitumor drug sensitivity. Furthermore, we established a novel association between GPC3 expression and cholesterol levels, validating GPC3’s functional role in gastric cancer biology. These findings provide new insights into the relationship between cholesterol metabolism and the tumor immune microenvironment, offering a robust framework for advancing individualized immunotherapy in gastric cancer.

## Materials and methods

### Data source and processing

Gene expression profiles along with the associated clinicopathological data for patients with gastric cancer were retrieved from the UCSC XENA database, specifically from the TCGA-STAD project (https://xenabrowser.net/datapages/). Additionally, the GSE62254 dataset was acquired from the Gene Expression Omnibus (GEO) repository (https://www.ncbi.nlm.nih.gov/geo/). This study encompasses a total of 675 samples, comprising 375 samples from the TCGA-STAD dataset and 300 from the GSE62254 dataset. To mitigate batch effects and ensure consistency across datasets, batch normalization was meticulously executed utilizing the ‘limma’ and ‘sva’ packages within the R statistical environment.

### Consensus molecular clustering based on cholesterol metabolism-related genes

We identified 50 cholesterol metabolism-related genes (CMRGs) from the Kyoto Encyclopedia of Genes and Genomes (KEGG) database (Pathway: hsa04979). Of these, 49 genes were found to be expressed in our integrated dataset. We employed the ‘‘ConsensusClusterPlus’’ (parameters: reps = 50, pItem = 0.8, pFeature = 1, distance = ‘educlidean’) R package for consensus clustering of the combined datasets, using expression levels of CMRGs. Pam and educlidean distances were adopted as the clustering algorithm and distance metric, respectively, with *k* = 3. To evaluate the independence of each gene isoform within the clusters, we conducted survival analysis and principal component analysis (PCA).

### Enrichment analysis and functional annotation

Gene Set Variation Analysis (GSVA) enrichment was performed to explore the heterogeneity of various biological processes using “GSVA” package. Hallmark gene sets “h.all.v2023.1.Hs.symbols.gmt”,KEGG gene sets “c2.cp.kegg.v2023.1.Hs.symbols.gmt”,Reactome gene sets “c2.cp.reactome.v2023.1.Hs.symbols.gmt”,wikipathways gene sets “c2.cp.wikipathways.v2023.1.Hs.symbols.gmt” and biocarta gene sets “c2.cp.biocarta.v2023.1.Hs.symbols.gmt” extracted from MSigDB database were used for GSVA. Compare the differences between pathways of 3 CholClusters, and the R package pheatmap can be used to generate separate heatmaps for the comparisons between three clusters. After identifying DEGs between CholClusters, GO and KEGG analysis was performed employing the clusterProfiler package.

### TME landscape analyses

To delineate variations in the tumor microenvironment (TME) across cholesterol subgroups, we initially conducted an analysis of stromal, immune, and ESTIMATE scores among the three subgroups utilizing the ESTIMATE algorithm. The infiltration levels of 23 immune cell types in each sample were quantified using the CIBERSORT algorithm. Furthermore, we applied single-sample gene set enrichment analysis (ssGSEA) to evaluate the relative abundance of immune cells within various clusters.

### Identification of DEGs and construction of geneClusters

Differentially expressed genes (DEGs) across the three CholClusters were identified utilizing the ‘limma’ package in R, applying criteria of an adjusted P value< 0.05 and a |logFC|>1. Survival-related DEGs were identified via univariate cox regression analysis with an P value <0.001, and patients with STAD were classified into two distinct geneClusters based on selected DEGs using R package “ConsensuClusterPlus”.

### Development of a CholScore prognostic system

We adopted the PCA algorithm to create a scoring system based on survival-related DEGs among the clusters in STAD named CholScore according to the formula: CholScore =∑(PC1 + PC2) where PC1 represents the largest proportion of the variance in the initial expression lineage to be decomposed, followed by PC2. All patients were classified into low-and high-CholScore groups at the optimal cutoff, which was calculated using the survminerR package. The Spearman correlation between CholScore and immune cells was analyzed.

### Stemness index calculation

This study employed the Stemness Index Workflow (https://bioinformaticsfmrp.github.io/PanCanStem_Web/) to calculate stemness indices (mRNAsi and mDNAsi), using a one-class algorithm based on a signature from normal stem cells. The mRNAsi is derived from a set of 11,774 genes, and the mDNAsi is based on differential methylation patterns across 151 CpG sites. Correlation between these indices and CholScore was analyzed.

### Mutation and drug sensitivity analysis

For the analysis of somatic mutations in stomach adenocarcinoma (STAD) across both low- and high-score groups, mutation annotation format (MAF) files were generated using the “maftools” package in R, with data from the TCGA database. We assessed the frequencies of copy number variations (CNVs), encompassing both gains and losses, and analyzed the proportion of single nucleotide variants (SNVs) utilizing GSCA database information. Furthermore, the tumor mutation burden (TMB) was computed for each STAD patient. The half-maximal inhibitory concentration (IC50) values of chemotherapeutic agents were determined using the “pRRophetic” package in R, drawing on drug data from the Genomics of Drug Sensitivity in Cancer (GDSC) database.

### Prediction of immunotherapy response

To validate the prediction of immunotherapy efficacy of the CholScore, three immunotherapeutic cohorts were used: melanoma treated with pembrolizumab, an anti-PD-1 antibody (GSE78220);melanoma treated with nivolumab, an anti-CTLA4 and anti-PD1 antibody(GSE91061); and advanced urothelial cancer treated with atezolizumab, an anti-PD-L1 antibody(IMvigor210 cohort).

### Cell lines and culture

The human gastric cancer (GC) cell line HGC27 was sourced from Zhong Qiao Xin Zhou Biotech, Shanghai, China, while the AGS cell line was procured from Cellcook Biotech, Guangzhou, China. The HGC27 cell culture was sustained in RPMI-1640 medium, acquired from Wisent, Shanghai, China, enriched with 10% fetal bovine serum (FBS, obtained from Wisent, Nanjing, China) and 1% penicillin/streptomycin (sourced from Thermo Fisher Scientific, Massachusetts, USA). Conversely, AGS cells were propagated in F-12K medium (Wisent, Nanjing, China), also supplemented with 10% FBS and 1% penicillin/streptomycin. Both cell lines were maintained under a controlled environment at 37°C, in a humidified atmosphere containing 5% CO2. Cholesterol and methyl-β-cyclodextrin (MβCD) were procured from Sigma-Aldrich, St. Louis, MO, USA.

### Tissue specimens

Sixty gastric cancer (GC) tissues were procured from patients undergoing surgical procedures at the First Affiliated Hospital of Nanjing Medical University (Nanjing, Jiangsu, China). All collected samples were promptly frozen in liquid nitrogen and preserved at ‐80°C until required. None of the GC patients had received preoperative chemotherapy or radiotherapy, and their diagnoses were confirmed through meticulous pathological analysis. Approval for this study was obtained from the Ethics Committee of the First Affiliated Hospital of Nanjing Medical University, and written consent was obtained from all participating patients.

### RNA isolation, reverse transcription, and quantitative PCR

Total RNA was extracted using TRIzol (ThermoFisher Scientific, USA) and reverse transcribed to cDNA from 500 ng RNA using the TRUEscriptRT kit (Proteinbio, China). qPCR was performed with SYBR Green Supermix (US EVERBRIGHT, China) on a 7500 Real-time PCR System (Applied Biosystems, USA) using specific primers (Proteinbio, China). mRNA levels were normalized to β-actin and analyzed by the 2−ΔΔCT method, with assays run in triplicate.

### RNA interference and plasmid transfection

To perform RNA interference, small interfering RNAs (siRNAs) specifically designed to target GPC3 were procured from GenePharma Biotechnology Co. Ltd (Shanghai, China). Briefly, approximately 6 × 10^5^ cells were plated in each well of a 6-well plate, reaching an approximate confluence of 80% after 24 hours in culture. Subsequently, a solution containing 50 nmol/L of the siRNA and 7.5 μL of Lipofectamine 3000 (Thermo Fisher Scientific, Massachusetts, USA) was prepared and allowed to incubate at room temperature for 20 minutes before application to the cells. Following a 48-hour incubation period, cells were collected for further analysis.

For plasmid transfection, plasmids encoding GPC3 and corresponding empty vectors were generated by Miaolingbio (Wuhan, China). Cells (6 × 10^5^) were seeded in a 6‐well plate and reached ∼80% confluence after 24 hours of culture. A mixture of 15 μg plasmid and 7.5 μL of Lipofectamine 3000 was incubated at room temperature for 20 minutes and then added to the cells. Cells were collected after 48 hours of culture.

### Immunohistochemical and hematoxylin and eosin staining

Immunohistochemistry (IHC) was conducted to assess GPC3 levels in the tumor tissue. Paraffin sections were deparaffinized, rehydrated, and antigen-retrieved with sodium citrate (10 mM, pH 6.0) using microwave treatment for 20 min. Endogenous peroxidase was blocked with 3% H2O2 for 10 min. Sections were blocked with 5% BSA, incubated with GPC3 antibody (Abcam, 1:100) at 37°C for 1 hr, then with HRP-linked secondary antibody (Abcam, 1:2000) at 37°C for 20 min. DAB and hematoxylin counterstaining were used for visualization.

For histopathological analysis, hematoxylin and eosin (H&E) staining was conducted on paraffin sections, which were deparaffinized, rehydrated, stained with hematoxylin for 5 min, differentiated in 1% hydrochloric acid alcohol, and stained with eosin for 2 min, followed by washing and water immersion for microscopic examination.

### Colony formation assay

Cells were plated in six‐well plates at a density of 500 cells per well and maintained in a complete medium for 10‐14 days. The resulting colonies were fixed with 4% PFA at room temperature for 15 minutes and subsequently stained with a 0.1% crystal violet solution for 30 minutes.

### Wound‐healing assay

Cells (6 × 10^5^/well) were cultured in 6-well plates until confluent, then wounded with a 200 µL pipette tip, and rinsed with PBS. Serum-free medium was used to inhibit proliferation. Wound healing was imaged at 0 and 24 hours, and areas analyzed with ImageJ. Experiments were performed in triplicate.

### Transwell assay

Transwell assays assessed migration/invasion, placing 3 × 10^4^ cells in serum-free medium in the upper chamber and 10% FBS medium (750 µL) in the lower chamber. After 36 hours, chambers were fixed with 4% PFA for 30 minutes, non-migrated cells removed, and migrated cells stained with 0.1% crystal violet for 20 minutes.

### Flow cytometric analysis of cell apoptosis

Apoptosis was assessed using an Annexin V-Alexa Fluor647 kit (US Everbright, Suzhou, China). Cells (6 × 10^5^) were cultured in 6-well plates for 24h, then stained with Annexin V-Alexa Fluor647 (50 µg/mL) and propidium iodide (10 µg/mL) for 15 min in darkness, followed by flow cytometry analysis (LSR, BD Biosciences, San Diego, CA, USA).

### Protein isolation and western blotting

GC cell lysates were prepared in RIPA buffer and protein concentration measured using a BCA kit (both from Beyotime, Haimen, China). Proteins were electrophoresed on 10% or 12.5% SDS-PAGE and transferred to PVDF membranes (Millipore, USA). Membranes were blocked, then incubated with primary antibodies (1:1000, ab95363, Abcam, USA) overnight at 4°C, followed by HRP-conjugated secondary antibodies for 1 hour at room temperature. After washing, protein detection was performed with a Super ECL kit (US Everbright, China), and quantification done using ImageJ (NIH, USA) as needed.

### Statistical analysis

Statistical analyses were conducted using R4.2.2 and GraphPad Prism 6.0, including Pearson and Spearman correlation, Kruskal-Wallis, and Pearson’s χ2 tests for group variations and categorical associations, respectively. Chi-square and Fisher’s exact tests were used for differential mutations and gene copy number analyses. Kaplan-Meier curves were generated using the “Survminer” R package. All tests were two-tailed with P < 0.05 significance.

## Results

### Identification of molecular subtypes in gastric cancer and construction of distinct CholClusters

**Figure 1:**
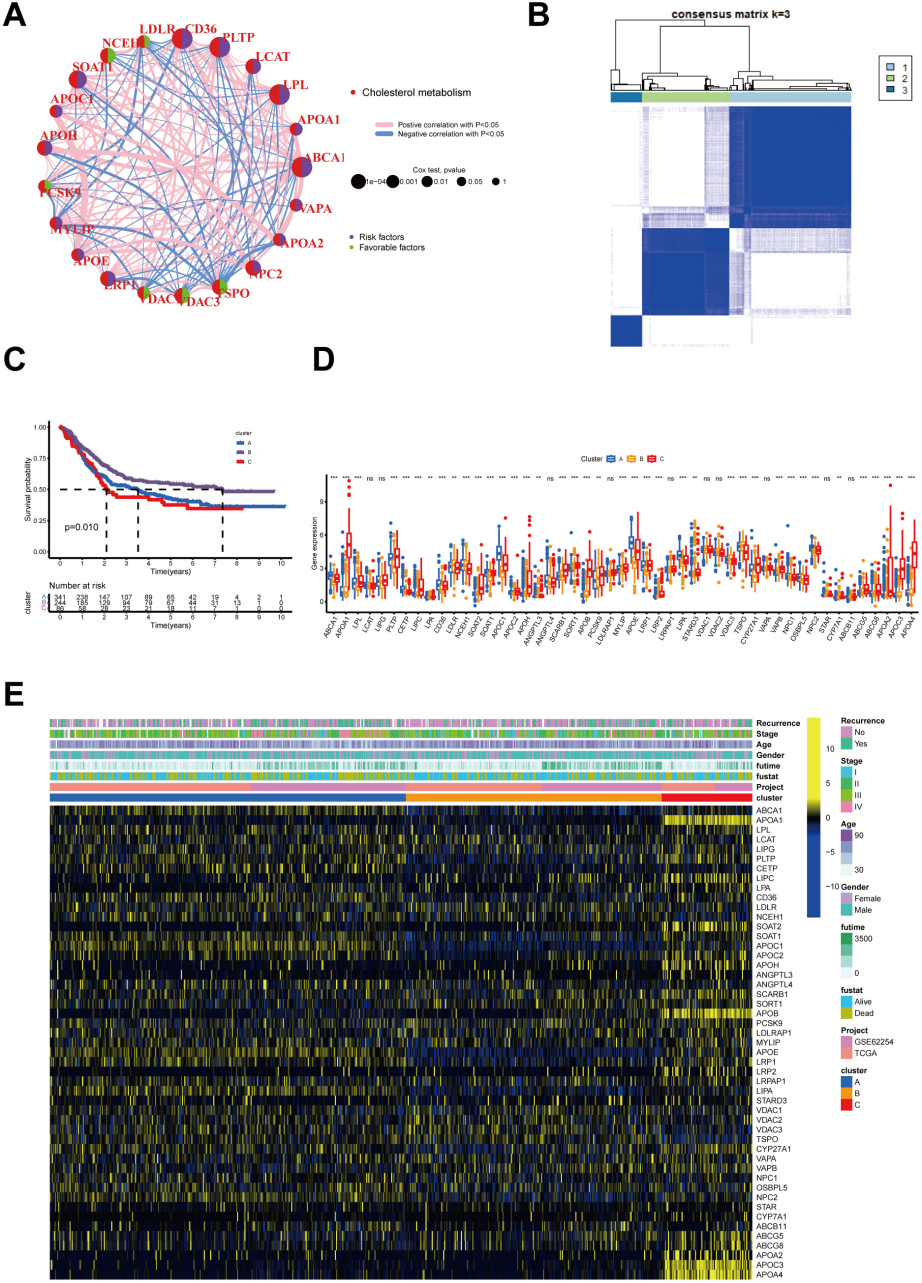
Interactions of Cholesterol metabolism-related genes and their prognostic value and identification of Cholesterol metabolism -associated cluster. **(A)** Interactions of Cholesterol metabolism regulators in GC. The circle diameter reflects the significance level of P values obtained from the Logrank test: p < 1e-04, p < 0.001, p < 0.01, p < 0.05, and p < 1. **(B)** The heatmap displays the consensus matrix obtained through consensus clustering with k = 3. **(C)** Significant variation in survival between the three Cholesterol metabolism-related patterns is depicted by Kaplan-Meier curves, with Log-rank p values < 0.05. **(D)** Differential expression of Cholesterol metabolism-related genes in three clusters (p>0.05, **p<0.01,***p<0.001). (E) Heatmap of genetic modification patterns. The term "ns" stands for "not significant," indicating that no statistically significant difference was observed.

Among the 50 genes related to cholesterol metabolism downloaded, 49 were found to be expressed in the merged dataset. A univariate Cox analysis was conducted on these genes, resulting in the identification of 21 cholesterol metabolism genes (**Supplementary Table S1**). We developed a CMRGs network to explore their interactions and prognostic value in gastric cancer (**Figure 1A**), finding 6 genes as favorable and 15 as risk factors. Furthermore, we conducted survival analysis by grouping the expression levels of CMRGs to further investigate their prognostic impact (**Supplementary Figure S1A**, displays genes with p<0.001).

**Figure 2:**
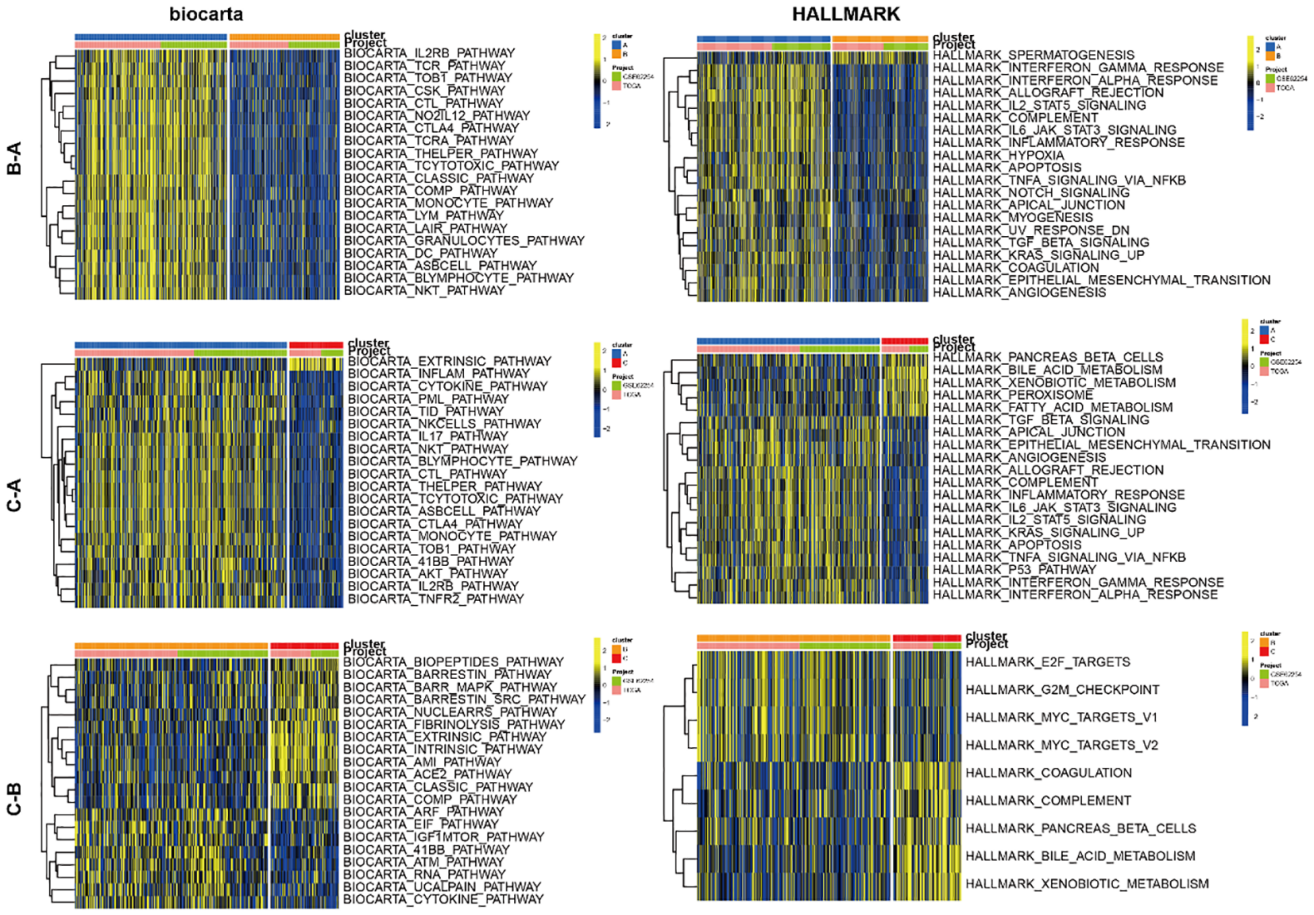
GSVA of biological pathways between three clusters.

To investigate the expression profiles of 49 CMRGs in gastric cancer, an unsupervised clustering analysis was performed using the R package “ ConsensusClusterPlus “ to classify individuals into three distinct types with the highest consensus, designated clusters A-C, respectively (**Figure 1B**). Cluster A includes 342 patients, Cluster B includes 246 patients, and Cluster C includes 87 patients. Survival analysis revealed that the overall survival of the three clusters was significantly different. CholCluster C had the worst prognosis in terms of overall survival, while CholCluster B had the best prognosis (**Figure 1C**). The three CholClusters showed significance in cholesterol metabolism-related genes’ expression(**Figure 1D**). Additionally, by integrating cholesterol subgroups with gastric cancer patients’ clinicopathological features into heatmaps of 49 cholesterol metabolism-related gene expressions, we observed higher expression levels in CholCluster C than in CholCluster A and B, suggesting more active cholesterol metabolism in Cluster C (**Figure 1E**). To delve deeper into the functional characteristics, our study utilized GSVA enrichment analysis with five comprehensive pathway databases (BioCarta, Reactome, Hallmark, WikiPathways, and KEGG). This analysis enabled us to identify and discern differential signaling pathways among the three clusters (**Figure 2**, **Supplementary Figures S1B, C**).

### Characteristics of the TME in three distinct CholClusters

**Figure 3:**
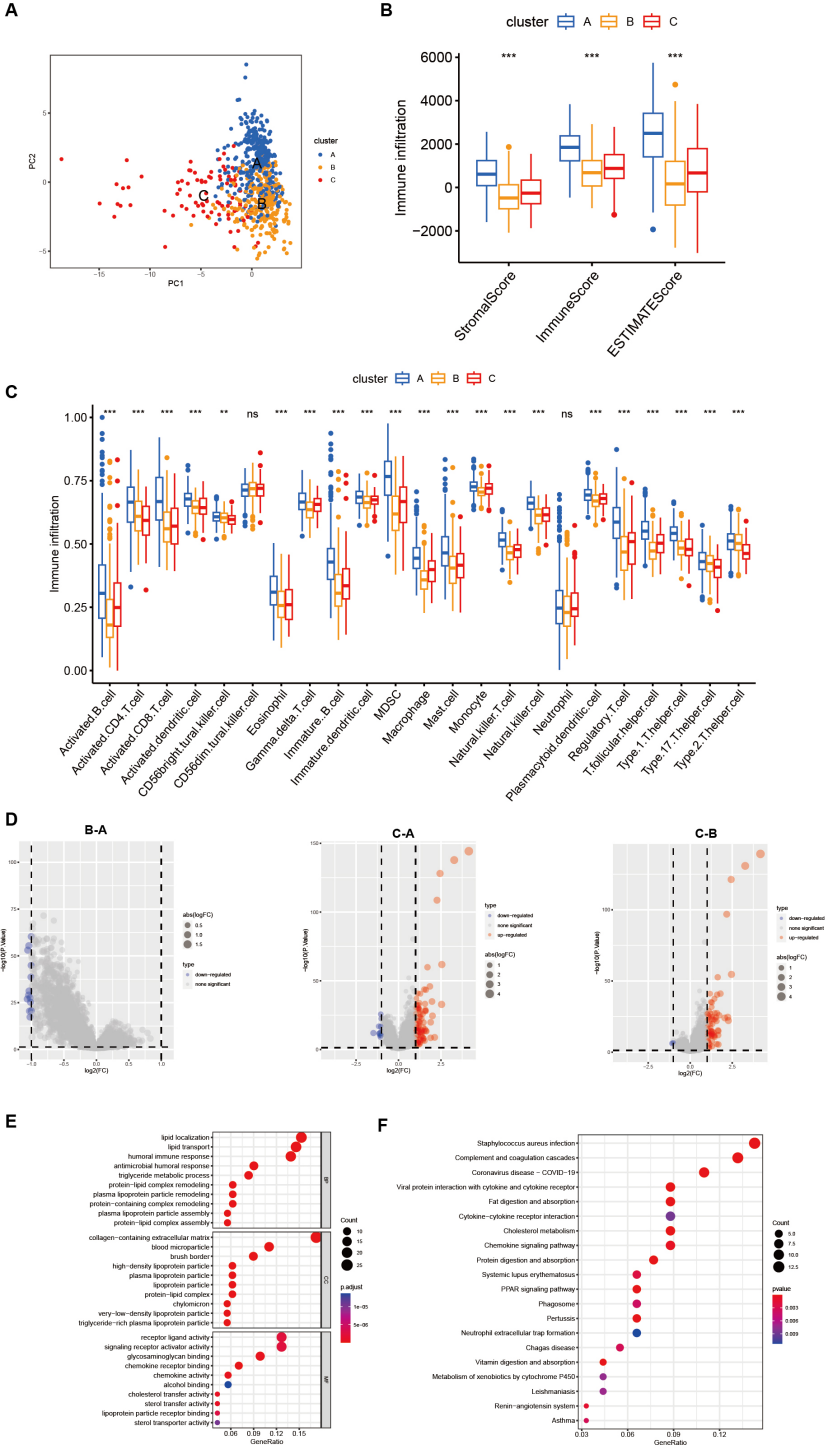
Differences in the tumor microenvironment between subgroups and functional enrichment analysis. **(A)** PCA shows different distributions between the three subgroups. **(B)** Stromal score, immune score, and ESTIMATE score analyses between three subgroups. **(C)** The abundance of 23 infiltrating immune cell types in the three different subgroups (ns p>0.05, **p<0.01, ***p<0.001). **(D)** Volcano plot show differential expressed genes in three clusters. **(E, F)** GO and KEGG enrichment analyses of DEGs among three subgroups.

While some overlap was observed, PCA analysis indicated a potential distinction between the three cholesterol metabolism modification patterns (**Figure 3A**). ESTIMATE analysis revealed TME differences among three clusters: Cluster A showed the highest stromal, immune, and ESTIMATE scores, with Cluster C and B following in order (**Figure 3B**). These results suggest that individuals with gastric cancer in Cluster A demonstrate heightened immune activity and reduced tumor purity. We also utilized the ssGSEA function from the R package GSVA to compute the scores of immune cell infiltration, thus comparing the disparities in immune cell infiltration among different subtypes. The results revealed that Cluster A was abundant in immune cell infiltrates, including eosinophil, MDSC, macrophage, mast cell, monocyte, natural killer cell and neutrophil (**Figure 3C**).

In order to further investigate the potential biological functions of each cholesterol metabolism pattern, we utilized the limma package to identify 150 differentially expressed genes (DEGs) associated with the cholesterol metabolism phenotype. A volcano plot was then generated (**Figure 3D**). Subsequently, we employed the R package clusterprofiler to perform Gene Ontology (GO) and Kyoto Encyclopedia of Genes and Genomes (KEGG) enrichment analysis on the DEGs. **Figure 3E** revealed that lipid localization, collagen-containing extracellular matrix and receptor ligand activity were the most enriched BP, CC and MF, respectively. The results of the KEGG enrichment analysis demonstrated that cholesterol metabolism-related DEGs are mainly enriched in pathways such as Staphylococcus aureus infection, complement and coagulation and coronavirus disease-COVID-19(**Figure 3F**).

Results showed that gastric cancer subtypes displayed diverse cholesterol metabolism levels, and cholesterol metabolism genes significantly influenced cell infiltration in the tumor microenvironment.

### Identification of cholesterol metabolism-related phenotypes and cholesterol scores

**Figure 4:**
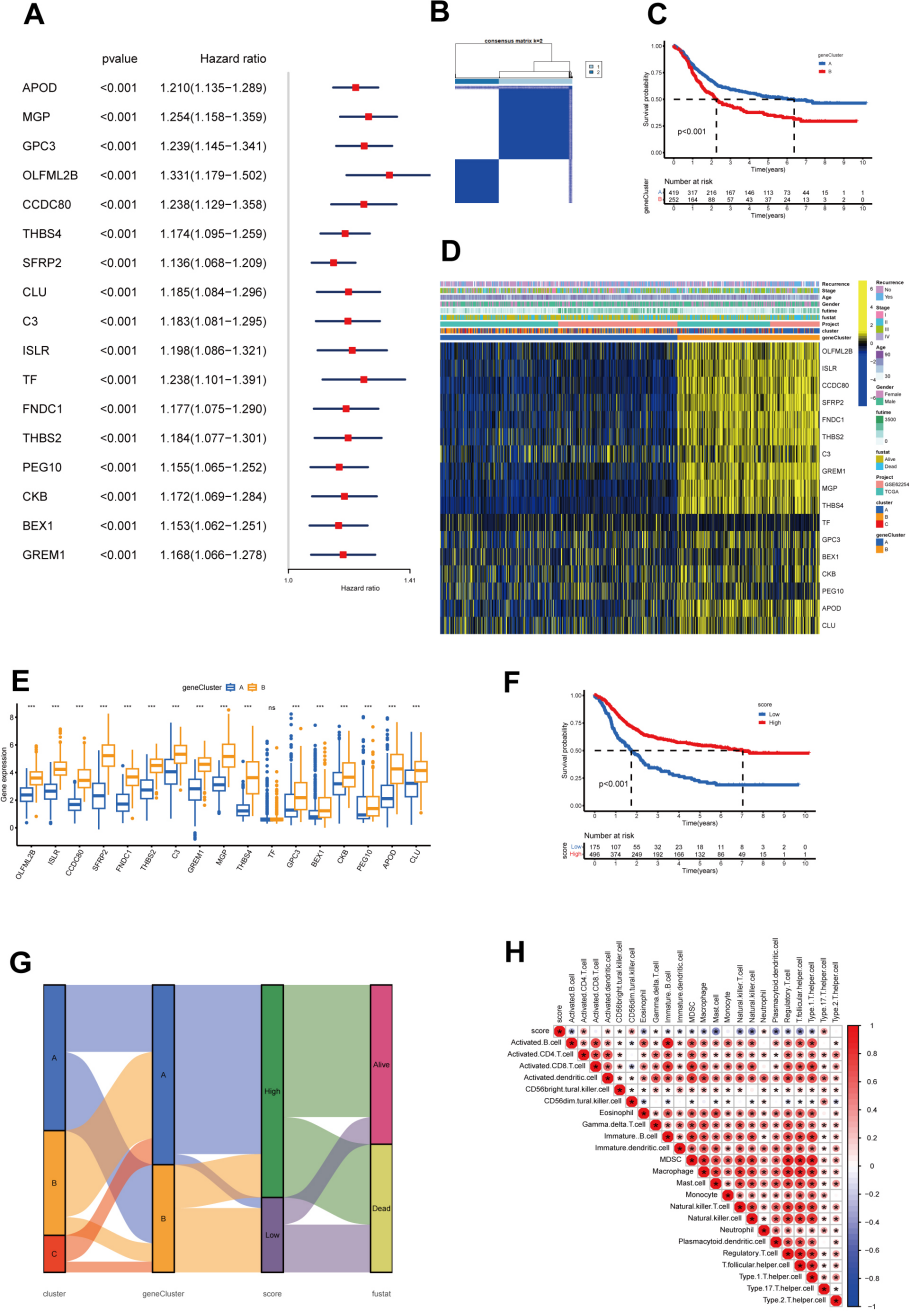
Construction of geneClusters and CholScores. **(A)** 17 genes exhibit a significant prognostic correlation identified through univariate Cox regression analysis, where the respective P-values are less than 0.001. **(B)** The heatmap representative of the consensus matrix generated by performing consensus clustering with a value of k = 2. **(C)** The top 12 genes with significant differences in mutation rates between the high and low-score groups, arranged in ascending order of p-values (*p<0.05, ***p<0.001). **(D)** A heatmap represents the expressions of 13 genes among the two gene subgroups, along with clinicopathological characteristics. **(E)** The differential expression patterns of the 13 genes within the two clusters (ns p>0.05, ***p<0.001). **(F)** The Kaplan-Meier curves for survival analysis carried out on both high and low-score groups. **(G)** A Sankey diagram illustrates the relationships among cluster, gene cluster, Cholscore, and survival status. **(H)** The relationship between immune cells and Cholscores, where red denotes a positive correlation, and blue denotes an inverse correlation (*p<0.05).

Next, the 150 DEGs related to cholesterol metabolism were examined using univariate Cox regression analysis to identify genes associated with overall survival (OS) in gastric cancer. We identified 17 genes significantly associated with the prognosis of gastric cancer patients (p < 0.001) (**Figure 4A**, **Supplementary Table S2**). To uncover the mechanisms behind prognostic DEGs in gastric cancer, we conducted unsupervised consensus clustering based on 17 prognostic genes, resulting in two distinct patient clusters: geneCluster A (422 cases) and geneCluster B (253 cases) (**Figure 4B**). Survival analysis demonstrated that the survival rate of geneClusterA was significantly higher than that of geneClusterB (**Figure 4C**). **Figure 4D** illustrates the relationship between the subtypes and clinical data. The expression levels of the majority of DEGs related to cholesterol metabolism were significantly upregulated in geneClusterB (**Figure 4E**).

To overcome the limitations of population-level analysis and accurately predict individual variations in cholesterol metabolism, we introduced a scoring system called the cholesterol metabolism score. The scoring system, based on PCA and phenotype-related genes, quantifies cholesterol metabolism patterns in gastric cancer. Survival analysis between high and low score groups indicated that higher scores were associated with better prognosis (**Figure 4F**). The Sankey diagram illustrates the relationship between subtypes, scores, and prognosis status (**Figure 4G**). We also examined the correlation between scores and immune cell infiltration (**Figure 4H**). The results revealed a positive correlation between the scores and several immune cells, such as activated CD4 cells and neutrophils. However, it showed a negative correlation with many immune suppressive cells, such as myeloid-derived suppressor cells (MDSCs) and regulatory T cells. This might be associated with the previously observed lower survival rates in the low-score group.

### The prognostic value of the cholesterol score in gastric cancer patients

**Figure 5:**
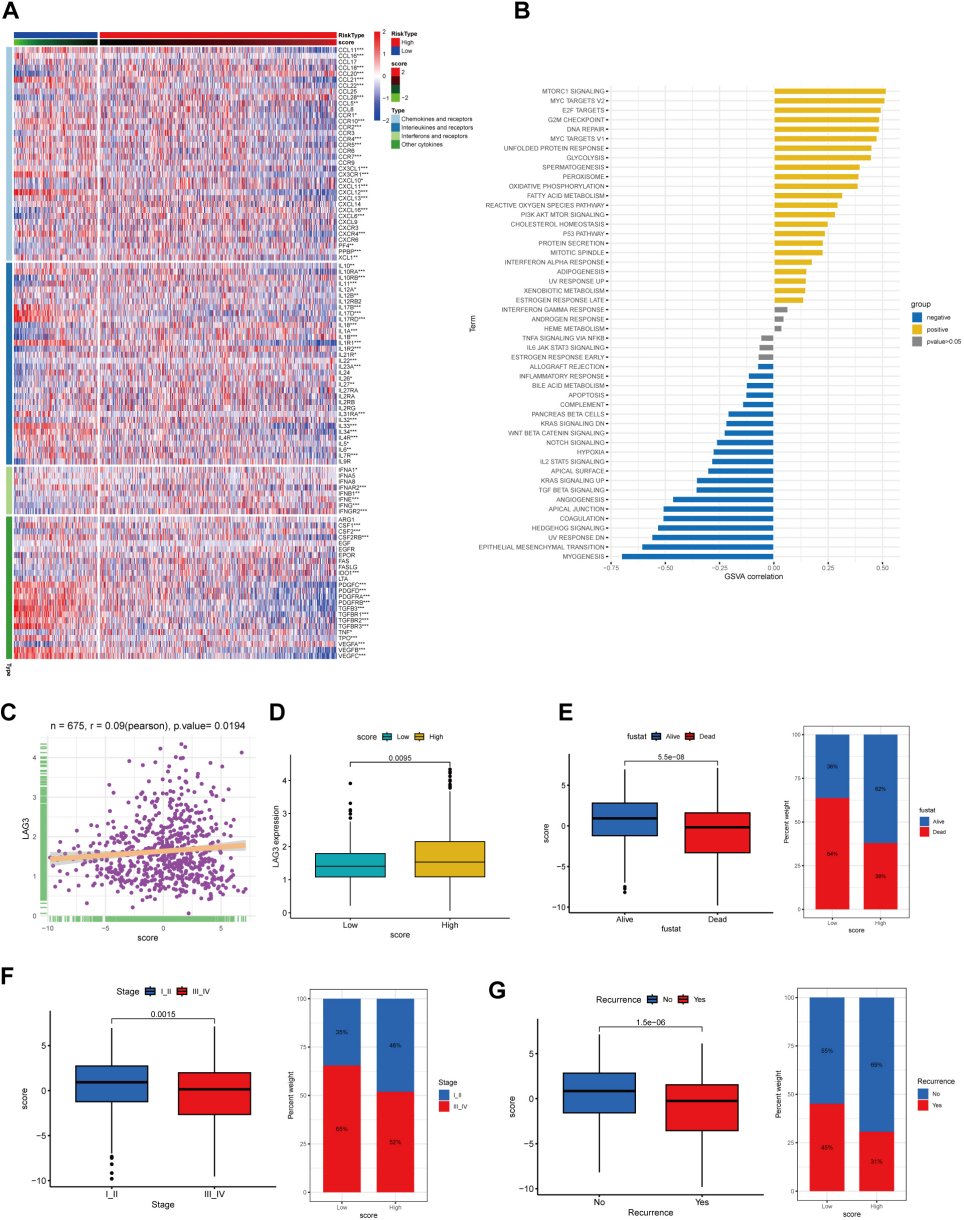
The correlation between CholScores and cytokines, along with GSVA analysis and assessment of clinical prognosis based on CholScores. **(A)** A heatmap depict the relationship between scores, chemokines, interleukins, interferons, their receptors, and other cytokines (p>0.05, *p<0.05,**p<0.01, ***p<0.001). **(B)** The correlation between scores and 50 hallmark pathways. **(C)** The relationship between LAG3 expression level and CholScores. **(D)** Differences in LAG3 expression between high and low CholScore groups. **(E)** Comparison of the CholScores between individuals in the Alive and Dead groups and examines the proportion of individuals with high and low CholScores in each group. **(F)** Comparison of the CholScores between individuals in the Stage I-II and Stage III-IV groups and examines the proportion of individuals in each high and low CholScore group within these stages. **(G)** Comparison of the CholScore between individuals in the Recurrence and No recurrence groups and examines the proportion of individuals in each high and low CholScore group within these recurrence categories.

Cytokines mediate key interactions between immune and non-immune cells in the tumor microenvironment (TME). **Figure 5A** demonstrates the heterogeneity in the relationship between the score and various chemokines, interleukins, interferons, their receptors, and other cytokines. It is worth noting that high scores are associated with upregulation of certain anti-tumor factors, such as IL18, while being correlated with downregulation of certain pro-oncogenic factors, such as IL33, IL4R, IL6, and IL34. Furthermore, in order to investigate the association between scores and the activity of hallmark pathways, we performed Gene Set Variation Analysis (GSVA) enrichment analysis. The GSVA analysis conducted on the hallmark pathways displayed an enrichment of multiple signaling pathways, including MTORC1 signaling, MYC targets V2, E2F targets, G2M checkpoint, and DNA repair, within the high-scoring group (**Figure 5B**).

Existing studies have shown that elevated LAG3 expression is unfavorable for the immunotherapeutic response in gastric cancer patients (14, 15). Therefore, we analyzed the correlation between LAG3 expression levels and cholesterol score, aiming to preliminarily explore the relationship between cholesterol score and the immunotherapeutic outcomes in gastric cancer patients. According to our findings, a higher score was associated with increased LAG3 expression levels, potentially indicating poorer immunotherapeutic efficacy in the high-score group of patients (**Figures 5C, D**).

We aimed to investigate the association between the cholesterol score and prognosis, as well as clinical characteristics. Results indicated that higher scores were associated with patient survival, early-stage (I-II) cancer, and absence of recurrence, correlating with lower mortality and recurrence rates (**Figures 5E–G**).

### Delineation of genome alterations among cholesterol metabolism phenotypes

**Figure 6:**
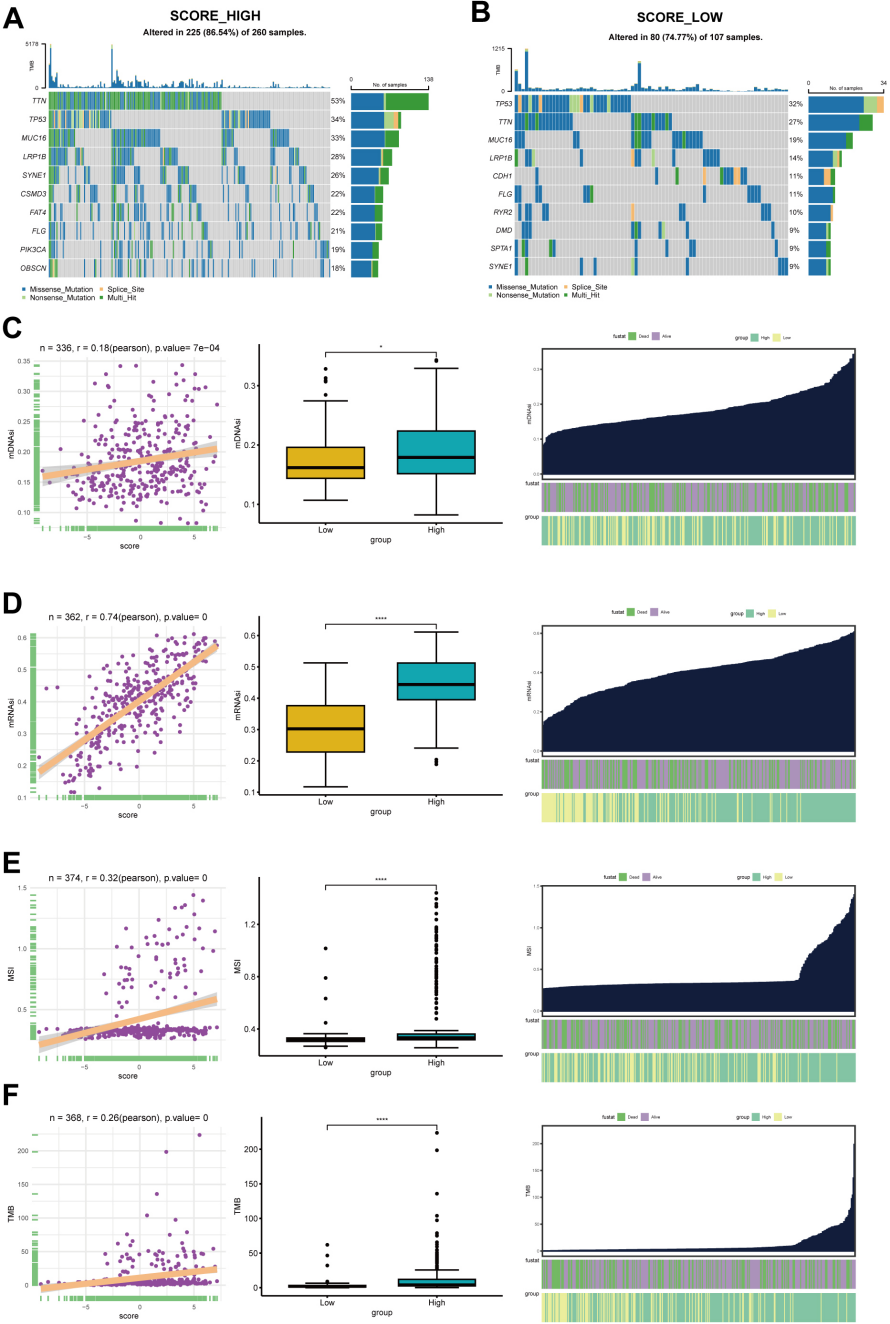
The somatic mutation analysis. **(A)** Waterfall plot depicts the top 20 genes with mutation frequency in the high-score group. **(B)** Waterfall plot shows the top 20 genes with mutation frequency in the low-score group. Each column in the plot represents an individual patient, with the upper histogram representing the total tumor mutation burden (TMB), and the numbers on the right indicating the mutation frequencies of each gene. The bar graph on the right represents the proportion of each mutation type. **(C)** The top 12 genes with significant differences in mutation rates between the high and low-score groups, arranged in ascending order of p-values (*p<0.05, ****p<0.0001). **(D)** The relationship between mDNAsi and CholScores, highlighting the differences in mDNAsi between the high and low groups. It also depicts the distribution of mDNAsi based on high vs low groups and patient survival status. **(E)** The relationship between mRNAsi and CholScores, displaying the differences in mRNAsi between the high and low groups. It further presents the distribution of mRNAsi based on high vs low groups and patient survival status. **(F)** The relationship between MSI and CholScores, highlighting the differences in mDNAsi between the high and low groups and the distribution of MSI based on high vs low groups and patient survival status. **(G)** The relationship between MSI and CholScores, highlighting the differences in mDNAsi between the high and low groups and the distribution of TMB based on high vs low groups and patient survival status.

Given the crucial role of TMB in guiding immunotherapy strategies for patients with STAD, we aimed to investigate the intrinsic relationship between TMB and cholesterol score, considering the clinical significance of TMB. Patients in the high-score group exhibited significantly elevated occurrences of somatic mutations in comparison with patients having high scores, particularly in the genes TTN (53% vs 27%), MUC16 (33% vs 19%), and LRP1B (28% vs 14%) (**Figures 6A, B**). **Figure 6C** illustrates a subset of genes that exhibit higher mutation rates in the high-score group.

The presence of CNV and SNV is a common and important hallmark of many cancers. In light of this, we investigated the CNV and SNV changes of screened cholesterol metabolism-related genes in further depth. Based on the pie chart of CNV distribution, the main types of CNV were heterozygous amplification (Hete Amp) or deletion (Hete Del) (**Supplementary Figure S2A**). PEG10 had the highest heterozygous amplification rate at 38.78% and the highest homozygous amplification rate at 5.9%, while SFRP2 showed the highest heterozygous deletion rate at 39.68%, and THBS2 had the highest homozygous deletion rate at 1.59% (**Supplementary Figure S2B**). Correlation analysis between CNV and mRNA expression levels reveals a positive association between the mRNA expression levels of THBS4, CKB, TF, and C3 genes and their copy number levels, while the mRNA expression level of OLFML2B demonstrates a negative correlation with their copy number levels (**Supplementary Figure S2C**). The methylation levels of the C3 gene demonstrated a positive correlation with mRNA expression levels, while the majority of the remaining genes exhibited a negative correlation between methylation levels and mRNA expression levels (**Supplementary Figure S2D**). As shown in **Supplementary Figure S3**, we also analyzed the SNV percentage of screened cholesterol metabolism-related genes, demonstrating that C3 gene had the highest SNV, followed by FNDC1, THBS2, OLFML2B, THBS4, CCDC80, TF, PEG10, ISLR, SFRP2, GPC3, CLU, APOD, CKB, MGP and BEX1(**Supplementary Figure S2E**).

The mRNAsi and the mDNAsi have been applied to assess cancer stem cell characteristics (14, 16). Our results demonstrate a positive correlation between cholesterol scores and both mRNAsi and mDNAsi. Moreover, the high-score group exhibited a higher stemness indexes (**Figures 6D, E**).The relationship between cholesterol scores and microsatellite instability (MSI) as well as tumor mutational burden (TMB) was also analyzed in this study. **Figures 6F, G** illustrate that higher cholesterol scores are associated with increased MSI and TMB levels. Previous studies have indicated that patients in the high-risk group exhibit higher TMB and MSI levels, along with a larger number of mutated genes. Therefore, we hypothesize that patients in the high-risk group may experience better therapeutic outcomes when undergoing immunotherapy. The aforementioned study suggests that cholesterol metabolism related genes are likely to impact tumor development through genetic and epigenetic modifications.

### Prediction of immunotherapy effectiveness and antitumor drug sensitivity among high- and low-scoring groups

**Figure 7:**
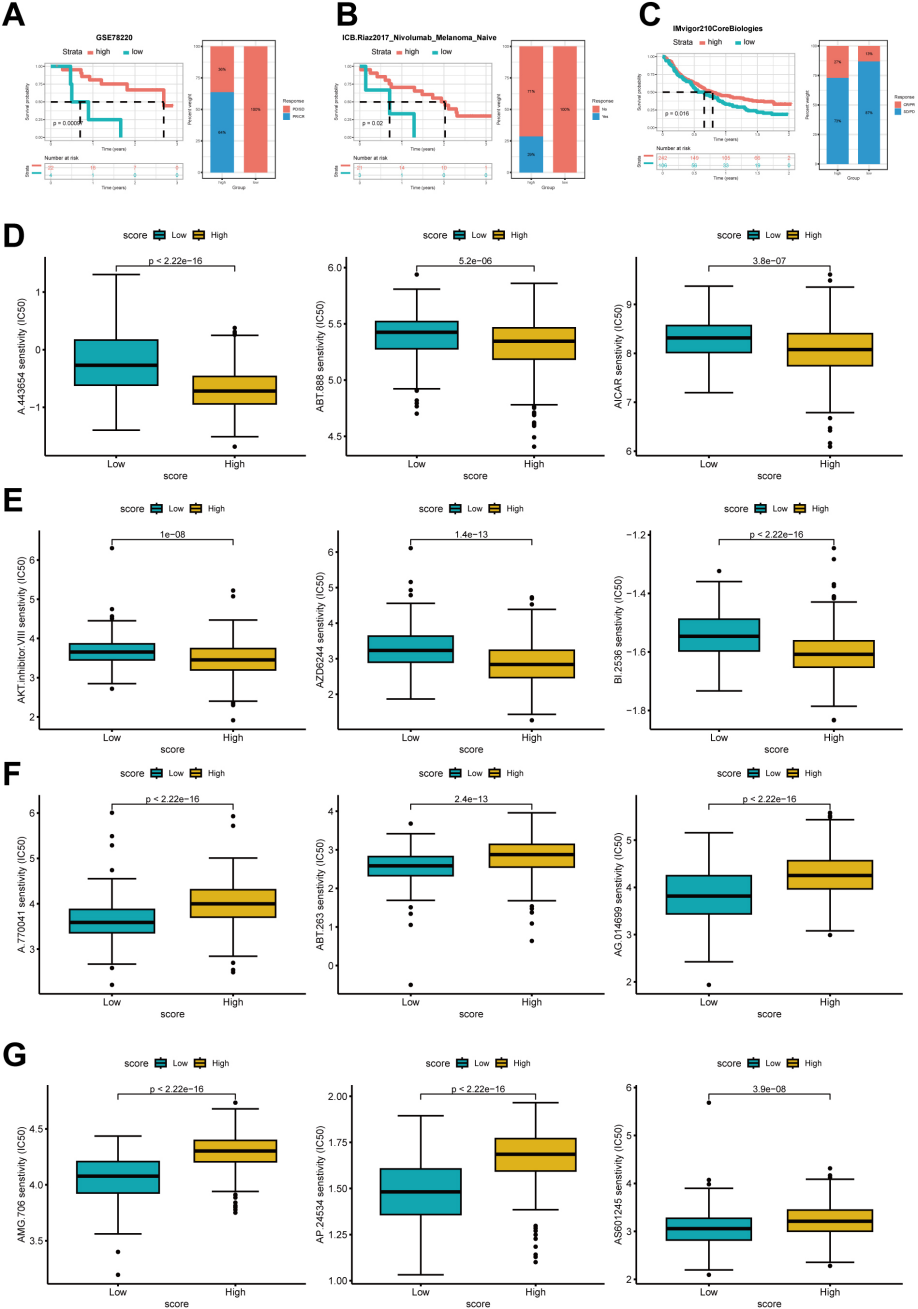
Prediction of immune therapy prognosis based on high and low groups. The Kaplan–Meier curve analysis of high- and low-score group in Anti-PD-1 cohorts and the proportions of PD/SD and PR/CR in the high and low groups **(A)**, the proportions of patients with responders and non-responders in each group **(B)**, and the proportions of patients with CR/PR and SD/PD **(C)**. **(D)** IC50 of several antitumor drugs in the high- and low score groups.IC50, half-maximal inhibitory concentration.

The aforementioned research demonstrates that the expression levels of cholesterol metabolism-related molecules are highly likely to contribute to the identification of tumor characteristics and offer novel therapeutic strategies. To thoroughly validate the predictive accuracy of the cholesterol score in assessing the efficacy of immunotherapy, we utilized various independent immunotherapy cohorts from the available literature for comprehensive assessment of both immunotherapy effectiveness and prognosis. The validation of the score’s predictive capacity for prognosis and immunotherapy efficacy was performed using pembrolizumab-treated melanoma, nivolumab-treated treatment-naive melanoma, and atezolizumab-treated advanced urothelial cancer (**Figures 7A–C**).

Compared to the low-scoring group, the high-scoring group showed significantly improved survival rates and enhanced responsiveness to immunotherapy.

We further investigated whether cholesterol scoring could accurately assess the chemotherapy sensitivity of gastric cancer patients. Ridge regression was employed to estimate the IC50 values in both the low-cholesterol and high-cholesterol groups for prediction purposes. Results showed that higher cholesterol score had lower IC50 of several drugs such as A.443654,ABT.888, AICAR,AKT inhibitor,AZD6244 and BI.2536. In contrast, patients with a low cholesterol score might respond more sensitively to A.770041,ABT.263,AG.014699,AMG.706,AP.24534 and AS6601245 (**Figure 7D**).

In summary, this study establishes a robust and significant association between the cholesterol modification pattern and the response to immune therapy in gastric cancer. Moreover, the developed cholesterol profile shows potential in predicting the responsiveness of gastric cancer patients to both immune therapy and other anti-cancer medications.

### The expression of GPC3 was significantly upregulated in high-cholesterol gastric cancer

**Figure 8:**
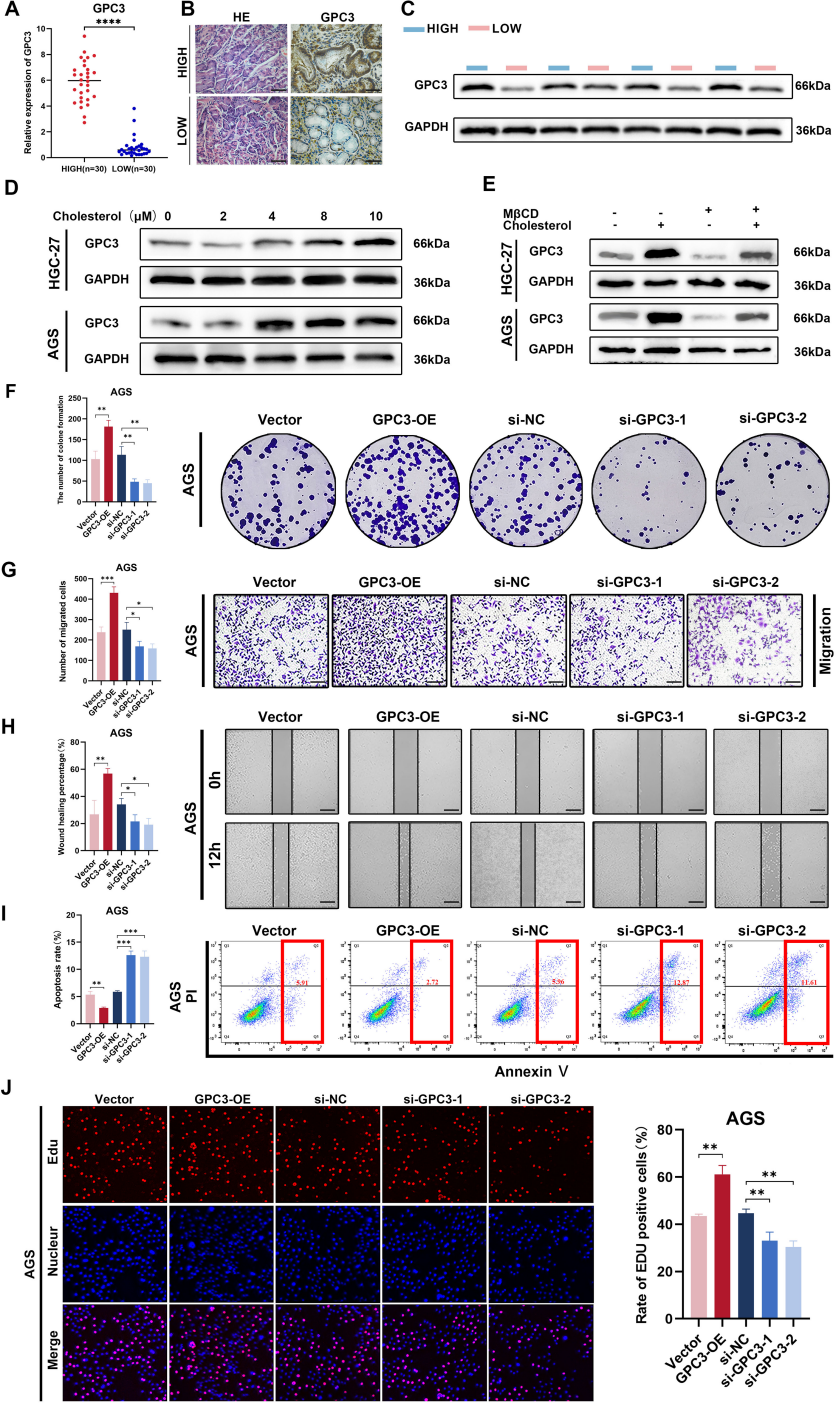
The expression and biological function of GPC3. **(A)** GPC3 level of 30 pairs high and low cholesterol GC tissues quantified by qRT-PCR. **(B)** IHC staining for GPC3 in high and low cholesterol GC tissues, scale bar = 100 μm. **(C)** Protein level of GPC3 was detected by western blot using 4 high-cholesterol GC tissues and 4 low-cholesterol GC tissues. **(D)** In the cell culture media of AGS and HGC-27, exogenous cholesterol was added at concentrations of 0, 2, 4, 8, and 10μM, respectively. After 24 hours of incubation, cell proteins were extracted, and the expression levels of GPC3 protein were assessed using Western blot analysis. **(E)** Cells were treated with 2.5 mM MβCD, 10 µM cholesterol or 2.5 mM MβCD +10 µM cholesterol for 24h, then GPC3 protein level in indicated cells was determined Western blot assay. **(F)** Colony formation assay was performed to evaluate proliferation ability of cells after upregulating or downregulating GPC3 in AGS cells. **(G)** Transwell assay was performed utilizing indicated engineered AGS cells. Scale bar: 100 μm. **(H)** Wound-healing assay was performed utilizing indicated engineered AGS cells. Scale bar: 200 μm. **(I)** Flow cytometric analysis revealed that overexpression of GPC3 inhibited apoptosis in AGS cells, while silencing of GPC3 had the opposite effect. **(J)** EdU assay was performed utilizing indicated engineered AGS cells. Error bars represent the mean (n = 3) ± S.D (p>0.05, *p<0.05, **p<0.01, ***p<0.001, **** p<0.0001).

We measured the cholesterol expression levels in the gastric cancer tissues of 60 patients and divided them into high and low cholesterol groups based on the median cholesterol expression levels in the tissues. Tissue proteins were extracted, and the mRNA expression levels of 17 genes between the high and low cholesterol groups were preliminarily validated through PCR (**Figure 8A**, **Supplementary Figure S3A**). The results showed significant statistical differences in the expression of six genes, namely GPC3, APOD, BEX1, CLU, GREM1, and PED10, between the high and low cholesterol groups, with GPC3 exhibiting the most significant difference. Recent studies have reported GPC3 as a crucial biomarker and therapeutic target for PD-1 blockade sensitivity in gastric cancer, sparking our interest (17). Consequently, we selected GPC3 for further investigation to explore its role in the development of high cholesterol gastric cancer. We randomly selected gastric cancer tissues from 5 patients in each group and performed consecutive sections. H&E and immunohistochemistry experiments were conducted on the tissue sections of these five pairs of patients to verify the differential expression of GPC3 between the two groups (**Figure 8B**). Additionally, we randomly selected gastric cancer tissues from 4 patients in each group and performed Western blotting to validate the protein-level expression of GPC3 (**Figure 8C**). The results indicated that GPC3 was highly expressed in gastric cancer tissues with high cholesterol.

To investigate whether the expression level of GPC3 in the tumor environment is correlated with cholesterol concentration, we selected two gastric cancer cell lines, HGC-27 and AGS. We simulated different cholesterol expressions in gastric cancer TME by adding exogenous cholesterol to the cell culture medium. The results showed that with increasing cholesterol concentration, the expression of GPC3 in AGS cell line exhibited an ascending trend, reaching its peak at 8μM, followed by stabilization. In the HGC-27 cell line, GPC3 expression showed an ascending trend, reaching its peak at 10μM (**Figure 8D**). Several studies have reported the application of MβCD for cholesterol removal (18, 19). Therefore, we chose MβCD to counteract the effect of cholesterol. The results showed that by adding MβCD, the expression of GPC3 was significantly down-regulated (2.5mM MβCD + 10μM cholesterol) (**Figure 8E**).

### GPC3 promotes malignant biological functions in gastric cancer

To explore whether GPC3 alters the biological functions of GC, we initially constructed two small interfering RNA (siRNA) molecules targeting GPC3. The effectiveness of siRNAs and the overexpression plasmid of GPC3 were validated through real-time quantitative PCR and Western blotting (**Supplementary Figures S3B, C**). Subsequently, functional gain or loss experiments were conducted. The results indicated that when GPC3 was overexpressed, the proliferation and migration of gastric cancer cells were significantly enhanced, while downregulation of GPC3 expression reduced the proliferation and migration capabilities of gastric cancer cells (**Figures 8F–H**, **Supplementary Figures S4A–C**). EdU experiments further demonstrated a significant reduction in cell proliferation ability in the si-GPC3-1 and si-GPC3-2 groups (**Figure 8J**, **Supplementary Figure S4E**). Additionally, flow cytometry analysis revealed that GPC3 overexpression markedly decreased the rate of cell apoptosis, enhancing the anti-apoptotic capabilities of GC cells (**Figure 8I**, **Supplementary Figure S4G**).

## Discussion

Gastric cancer (GC) is a leading cause of cancer-related mortality globally, ranking fifth in incidence (5.6%) and third in mortality (7.7%) among malignant tumors (1, 20). Due to its high incidence and rapid progression, fewer than 30% of GC cases are diagnosed at an early stage, often resulting in metastasis by the time of diagnosis (21). With the increasing use of immunotherapy in advanced GC, it is crucial to develop tools for predicting prognosis and assessing treatment efficacy. Cholesterol is a key component of cell membranes, and its synthesis and accumulation are essential for cell proliferation. Dysregulation of cholesterol metabolism, involving metabolites like mevalonate, isoprenoids, ubiquinone, and sterols, has been linked to cancer pathogenesis (22). Recent studies show that activation of the IRE1α/XBP1 pathway in tumor cells promotes tumor growth by enhancing cholesterol synthesis and secretion, while suppressing anti-tumor immunity (23). Therefore, this study aims to explore the role of cholesterol metabolism in the tumor microenvironment, prognosis, and the efficacy of chemotherapy and immunotherapy in GC.

In this study, based on 49 cholesterol metabolism-related genes, we classified all gastric cancer patients into three clusters with significant differences in prognosis, enriched pathways, and distinct tumor microenvironments. Cluster B exhibited the highest survival rate, followed by Cluster A and Cluster C. Differential expression analysis of the 49 cholesterol metabolism genes showed that the majority of them had higher expression levels in Cluster C. GSVA analysis indicated increased activity in cholesterol metabolism pathways, bile acid metabolism pathways, fatty acid metabolism pathways, and metabolism of steroids pathways in Cluster C, suggesting a more active cholesterol metabolism in this cluster. The ssGSEA analysis revealed that the infiltration levels of numerous immune cells were relatively low in Cluster C. This is consistent with the findings of many previous studies, which suggest that cholesterol and its metabolites act as signaling molecules that promote tumor progression, and that cholesterol plays a significant role in dampening anti-tumor immune responses (12). It is worth noting that some tumor-related signaling pathways are relatively active in Cluster C, such as the MAPK pathway. Previous studies have indicated a close association between the MAPK signaling pathway and the proliferation and occurrence of gastric cancer (24–26). In addition, Chan, L. K. et al. found that activation of the MAPK signaling pathway enhances the expression of cholesterol biosynthesis-related genes in HCC (hepatocellular carcinoma) cells (27). Therefore, we can speculate that cholesterol metabolism may be closely associated with the MAPK pathway in the development and progression of gastric cancer. This provides a new avenue for further research into the pathophysiology of gastric cancer and the exploration of novel treatment approaches.

To evaluate the clinical relevance of this subtyping, we identified 17 DEGs associated with gastric cancer prognosis and performed unsupervised clustering, categorizing patients into two subtypes. A cholesterol score was then developed based on gene expression levels to guide immunotherapy and address individual heterogeneity. We analyzed the correlation between the cholesterol score and immune-related cytokines, including chemokines, interleukins, interferons, and their receptors. The high-score group showed elevated CXCL10 expression, while the low-score group exhibited higher levels of CXCL12, CXCR4, IL33, and TGF-β. Studies have indicated that CXCL10 may promote the expression of IFN signature genes and TAA, potentially facilitating T cell infiltration into the tumor, ultimately enhancing PD-L1 and PD-1 blockade therapy (28). The CXCL12-CXCR4 axis hinders T cell infiltration into the tumor, thereby enhancing resistance to immune checkpoint inhibitors (ICIs) (12). In the context of the B16F10 melanoma model, a specific IL-33-blocking antibody has shown promise in restoring the effectiveness of anti-PD1 therapy, especially in subclones exhibiting resistance to ICIs (24). Additionally, elevated levels of TGF-β are frequently linked to diminished responsiveness to PD-1/PD-L1 therapy (25). As a result, we speculate that patients in the high-score group may potentially benefit more from immunotherapy. Subsequently, we conducted an analysis of somatic cell mutations, tumor mutation burden, and microsatellite instability (MSI) between the high and low-score groups. The results indicated that the high-score group exhibited higher somatic cell mutation rates, tumor mutation burden, and MSI. Ke, L., S. Li, et al. (29) found in gastric cancer that the level of tumor mutation burden is positively correlated with the effectiveness of immune checkpoint inhibitors (ICIs). Furthermore, MSI is also considered a predictive biomarker for immunotherapy response, which aligns with our previous speculation (30–33). We also incorporated data from three datasets, including patients receiving immunotherapy and those not receiving immunotherapy, to validate the specific significance of the cholesterol score in immunotherapy prediction. Clearly, patients in the high-score group benefit more from immunotherapy. Statins have been shown to improve survival and reduce mortality in metastatic cancer patients (34). Given the impact of cholesterol metabolism on immune cells, drugs targeting cholesterol pathways may influence immunotherapy outcomes. By inhibiting cholesterol synthesis and lowering blood cholesterol levels, statins could potentially enhance the efficacy of immunotherapy (35). Our study utilized a cholesterol scoring system to predict the effectiveness of anti-tumor drugs in high- and low-score groups. Further clinical studies incorporating statin co-treatment may help refine and optimize this system. Due to the influence of cholesterol metabolism on immune cells, drugs that act on cholesterol metabolism may also affect the efficacy of immunotherapy.

We measured cholesterol levels in gastric cancer tissues from 60 patients, dividing them into high- and low-cholesterol groups based on the median expression. PCR analysis of 17 genes revealed GPC3 as the most significantly differentially expressed gene.GPC3 is a membrane-associated proteoglycan that is specifically up-regulated in hepatocellular carcinoma (HCC) (36). As a biomarker for HCC, GPC3 has been extensively studied as a novel therapeutic target. However, research on GPC3 in GC remains limited. LI D et al. found GPC3 is a critical biomarker and therapeutic target for sensitizing the PD-1 blockage therapy in GC (37). In this study, we confirmed through *in vitro* cell experiments that cholesterol promotes the expression of GPC3 in gastric cancer cells. Additionally, we verified that the expression of GPC3 is to some extent dependent on the concentration of cholesterol and identified its function in gastric cancer cells. Previous studies have indicated that GPC3 functions as an oncofetal antigen, contributing to Wnt-dependent cell proliferation (38). Moreover, cholesterol has been shown to enhance tumor growth through the Fzd5-mediated Wnt/β-catenin signaling pathway (19). Therefore, we hypothesize that cholesterol may influence the expression and function of GPC3 through the Wnt pathway. The specific molecular mechanisms await further investigation.

In this study, the cholesterol score in GC was strongly associated with tumor mutational load, genomic instability, immune cell infiltration, immune evasion, and response to ICI treatment. These findings offer new perspectives for GC diagnosis and therapy, highlighting CMRGs as potential biomarkers or therapeutic targets. However, this study has limitations. First, the metabolic classifications were derived from public datasets, and their clinical relevance requires further validation. Second, while we uncovered a novel association between GPC3 expression and cholesterol levels and confirmed GPC3’s role in GC biology, the underlying immune modulatory mechanisms remain unclear and warrant further investigation, such as sequencing analysis. Lastly, the effects of CMRGs on immune cell infiltration and genomic instability in GC need deeper exploration.

In conclusion, the cholesterol score we developed can improve chemotherapy selection for GC patients and serve as a valuable tool for predicting immunotherapy efficacy.

## Conclusion

Our research findings indicate a significant improvement in survival rates in the high-score subgroup compared to the low-score subgroup. The high-score subgroup exhibits higher levels of TMB and MSI, as well as a greater number of mutated genes. This high-score subgroup demonstrates enhanced sensitivity to immunotherapy. The significance of this prognostic score was validated through real-world cohorts of patients undergoing immunotherapy. Finally, we confirmed the correlation between GPC3 expression levels and cholesterol concentration, validating the biological functionality of GPC3. These research findings underscore the critical role of CMRGs, deepen our understanding of the tumor immune microenvironment, and provide guidance for personalized immunotherapy for gastric cancer patients.

## Data Availability

The datasets presented in this study can be found in online repositories. The names of the repository/repositories and accession number(s) can be found in the article/**Supplementary Material**.

## References

[1] Ajani JA D'Amico TA Bentrem DJ Chao J Cooke D Corvera C. Gastric cancer, version 2.2022, NCCN clinical practice guidelines in oncology. J Natl Compr Canc Netw. (2022) 20:167–92. doi: 10.6004/jnccn.2022.0008 35130500

[2] Xu Z Wang L Dai S Chen M Li F Sun J. Epidemiologic trends of and factors associated with overall survival for patients with gastroenteropancreatic neuroendocrine tumors in the United States. JAMA Netw Open. (2021) 4:e2124750. doi: 10.1001/jamanetworkopen.2021.24750 34554237 PMC8461504

[3] Li Y Feng A Zheng S Chen C Lyu J. Recent estimates and predictions of 5-year survival in patients with gastric cancer: A model-based period analysis. Cancer Control. (2022) 29:10732748221099227. doi: 10.1177/10732748221099227 35499497 PMC9067041

[4] Wang D-K Zuo Q He Q-Y Li B. Targeted immunotherapies in gastrointestinal cancer: from molecular mechanisms to implications. Front Immunol. (2021) 12:705999. doi: 10.3389/fimmu.2021.705999 34447376 PMC8383067

[5] Ying L Ferrero RL. Role of NOD1 and ALPK1/TIFA signalling in innate immunity against helicobacter pylori infection. Curr Top Microbiol Immunol. (2019) 421:159–77. doi: 10.1007/978-3-030-15138-6_7 31123889

[6] Shang W Liang X Li S Li T Zheng L Shao W. Orphan nuclear receptor Nurr1 promotes Helicobacter pylori-associated gastric carcinogenesis by directly enhancing CDK4 expression. EBioMedicine. (2020) 53:102672. doi: 10.1016/j.ebiom.2020.102672 32114387 PMC7047206

[7] Pih GY Gong EJ Choi JY Kim M Ahn JY Choe J. Associations of serum lipid level with gastric cancer risk, pathology, and prognosis. Cancer Res Treat. (2021) 53:445–56. doi: 10.4143/crt.2020.599 PMC8053878 33253515

[8] Shen JG Jin L Dong M Wang L Zhao W Shen J. Low level of serum high-density lipoprotein cholesterol in gastric cancer correlates with cancer progression but not survival. Transl Cancer Res. (2020) 9:6206–13. doi: 10.21037/tcr-20-1220 PMC8798992 35117231

[9] Huang B Song B-L Xu C. Cholesterol metabolism in cancer: mechanisms and therapeutic opportunities. Nat Metab. (2020) 2:132–41. doi: 10.1038/s42255-020-0174-0 32694690

[10] Xu H Zhou S Tang Q Xia H Bi F. Cholesterol metabolism: New functions and therapeutic approaches in cancer. Biochim Biophys Acta Rev Cancer. (2020) 1874:188394. doi: 10.1016/j.bbcan.2020.188394 32698040

[11] Wang S Yao Y Rao C Zheng G Chen W. 25-HC decreases the sensitivity of human gastric cancer cells to 5-fluorouracil and promotes cells invasion via the TLR2/NF-κB signaling pathway. Int J Oncol. (2019) 54:966–80. doi: 10.3892/ijo.2019.4684 PMC6365050 30664194

[12] Ma X Bi E Lu Y Su P Huang C Liu L. Cholesterol induces CD8+ T cell exhaustion in the tumor microenvironment. Cell Metab. (2019) 30:43–156.e145. doi: 10.1016/j.cmet.2019.04.002 PMC7061417 31031094

[13] Ning Y Fang S Zhang R Fang J Lin K Ding Y. Simvastatin induces ferroptosis and activates anti-tumor immunity to sensitize anti-PD-1 immunotherapy in microsatellite stable gastric cancer. Int Immunopharmacol. (2024) 142:113244. doi: 10.1016/j.intimp.2024.113244 39317047

[14] Pan S Zhan Y Chen X Wu B Liu B. Identification of biomarkers for controlling cancer stem cell characteristics in bladder cancer by network analysis of transcriptome data stemness indices. Front Oncol. (2019) 9:613. doi: 10.3389/fonc.2019.00613 31334127 PMC6620567

[15] Kim R An M Lee H Mehta A Heo Y J Kim KM. Early tumor-immune microenvironmental remodeling and response to first-line fluoropyrimidine and platinum chemotherapy in advanced gastric cancer. Cancer Discov. (2022) 12:984–1001. doi: 10.1158/2159-8290.CD-21-0888 PMC9387589 34933901

[16] Malta TM Sokolov A Gentles AJ Burzykowski T Poisson L Weinstein JN. Machine learning identifies stemness features associated with oncogenic dedifferentiation. Cell. (2018) 173:338–54.e15. doi: 10.1016/j.cell.2018.03.034 PMC5902191 29625051

[17] Tang W Li G Lin Q Zhu Z Wang Z Wang Z. Multiplex immunohistochemistry defines two cholesterol metabolism patterns predicting immunotherapeutic outcomes in gastric cancer. J Transl Med. (2023) 21:887. doi: 10.1186/s12967-023-04758-4 38062450 PMC10702056

[18] Pan Z Wang K Wang X Jia Z Yang Y Duan Y. Cholesterol promotes EGFR-TKIs resistance in NSCLC by inducing EGFR/Src/Erk/SP1 signaling-mediated ERRα re-expression. Mol Cancer. (2022) 21:77. doi: 10.1186/s12943-022-01547-3 35303882 PMC8932110

[19] Zheng S Lin J Pang Z Zhang H Wang Y Ma L. Aberrant cholesterol metabolism and wnt/β-catenin signaling coalesce via frizzled5 in supporting cancer growth. Adv Sci (Weinh). (2022) 9:e2200750. doi: 10.1002/advs.202200750 35975457 PMC9534957

[20] Geeleher P Cox N Huang RS. pRRophetic: an R package for prediction of clinical chemotherapeutic response from tumor gene expression levels. PloS One. (2014) 9:e107468. doi: 10.1371/journal.pone.0107468 25229481 PMC4167990

[21] Li K Zhang A Li X Zhang H Zhao L. Advances in clinical immunotherapy for gastric cancer. Biochim Biophys Acta Rev Cancer. (2021) 1876:188615. doi: 10.1016/j.bbcan.2021.188615 34403771

[22] Ding X Zhang W Li S Yang H. The role of cholesterol metabolism in cancer. Am J Cancer Res. (2019) 9:219–27. PMC6405981 30906624

[23] Yang Z Huo Y Zhou S Guo J Ma X Li T. Cancer cell-intrinsic XBP1 drives immunosuppressive reprogramming of intratumoral myeloid cells by promoting cholesterol production. Cell Metab. (2022) 34:2018–2035 e2018. doi: 10.1016/j.cmet.2022.10.010 36351432

[24] Du F Du F Sun L Chu Y Li T Lei C. DDIT4 promotes gastric cancer proliferation and tumorigenesis through the p53 and MAPK pathways. Cancer Commun (Lond). (2018) 38:45. doi: 10.1186/s40880-018-0315-y 29976242 PMC6034313

[25] Xiang Z Li J Song S Wang J Cai W Hu W. A positive feedback between IDO1 metabolite and COL12A1 via MAPK pathway to promote gastric cancer metastasis. J Exp Clin Cancer Res. (2019) 38:314. doi: 10.1186/s13046-019-1318-5 31315643 PMC6637527

[26] Wu S Chen M Huang J Zhang F Lv Z Jia Y. ORAI2 promotes gastric cancer tumorigenicity and metastasis through PI3K/akt signaling and MAPK-dependent focal adhesion disassembly. Cancer Res. (2021) 81:986–1000. doi: 10.1158/0008-5472.CAN-20-0049 33310726

[27] Chan LK Ho DW Kam CS Chiu EY Lo IL Yau DT. RSK2-inactivating mutations potentiate MAPK signaling and support cholesterol metabolism in hepatocellular carcinoma. J Hepatol. (2021) 74:360–71. doi: 10.1016/j.jhep.2020.08.036 32918955

[28] Nagarsheth N Wicha MS Zou W. Chemokines in the cancer microenvironment and their relevance in cancer immunotherapy. Nat Rev Immunol. (2017) 17:559–72. doi: 10.1038/nri.2017.49 PMC5731833 28555670

[29] Ke L Li S Huang D. The predictive value of tumor mutation burden on survival of gastric cancer patients treated with immune checkpoint inhibitors: A systematic review and meta-analysis. Int Immunopharmacol. (2023) 124:110986. doi: 10.1016/j.intimp.2023.110986 37748223

[30] Dudley JC Lin MT Le DT Eshleman JR. Microsatellite instability as a biomarker for PD-1 blockade. Clin Cancer Res. (2016) 22:813–20. doi: 10.1158/1078-0432.CCR-15-1678 26880610

[31] Hargadon KM Johnson CE Williams CJ. Immune checkpoint blockade therapy for cancer: An overview of FDA-approved immune checkpoint inhibitors. Int Immunopharmacol. (2018) 62:29–39. doi: 10.1016/j.intimp.2018.06.001 29990692

[32] Le DT Durham JN Smith KN Wang H Bartlett BR Aulakh LK. Mismatch repair deficiency predicts response of solid tumors to PD-1 blockade. Science. (2017) 357:409–13. doi: 10.1126/science.aan6733 PMC5576142 28596308

[33] Le DT Uram JN Wang H Li G. PD-1 blockade in tumors with mismatch-repair deficiency. N Engl J Med. (2015) 372:2509–20. doi: 10.1056/NEJMoa1500596 PMC4481136 26028255

[34] Hindler K Cleeland CS Rivera E Collard CD. The role of statins in cancer therapy. Oncologist. (2006) 11:306–15. doi: 10.1634/theoncologist.11-3-306 16549815

[35] Zhang H Zhao W Li X He Y. Cholesterol metabolism as a potential therapeutic target and a prognostic biomarker for cancer immunotherapy. Onco Targets Ther. (2021) 14:3803–12. doi: 10.2147/OTT.S315998 PMC8232957 34188488

[36] Zheng X Liu X Lei Y Wang G Liu M. Glypican-3: A novel and promising target for the treatment of hepatocellular carcinoma. Front Oncol. (2022) 12:824208. doi: 10.3389/fonc.2022.824208 35251989 PMC8889910

[37] Li D Wang Y Shi C Fu S Sun Y Li C. Targeting GPC3high cancer-associated fibroblasts sensitizing the PD-1 blockage therapy in gastric cancer. Ann Med. (2023) 55:2189295. doi: 10.1080/07853890.2023.2189295 37036308 PMC10088929

[38] Li D Li N Zhang Y Fu H Feng M Schneider D. Persistent polyfunctional chimeric antigen receptor T cells that target glypican 3 eliminate orthotopic hepatocellular carcinomas in mice. Gastroenterology. (2020) 158:2250–65.e20. doi: 10.1053/j.gastro.2020.02.011 PMC7282931 32060001

