# Supplementary figures and images for: Prediction of prognosis, immunogenicity and efficacy of immunotherapy based on cholesterol metabolism in gastric cancer
Source: Front Oncol. 2024 Dec 24;14:1518010. doi: 10.3389/fonc.2024.1518010 (PMC11703741; doi:10.3389/fonc.2024.1518010)

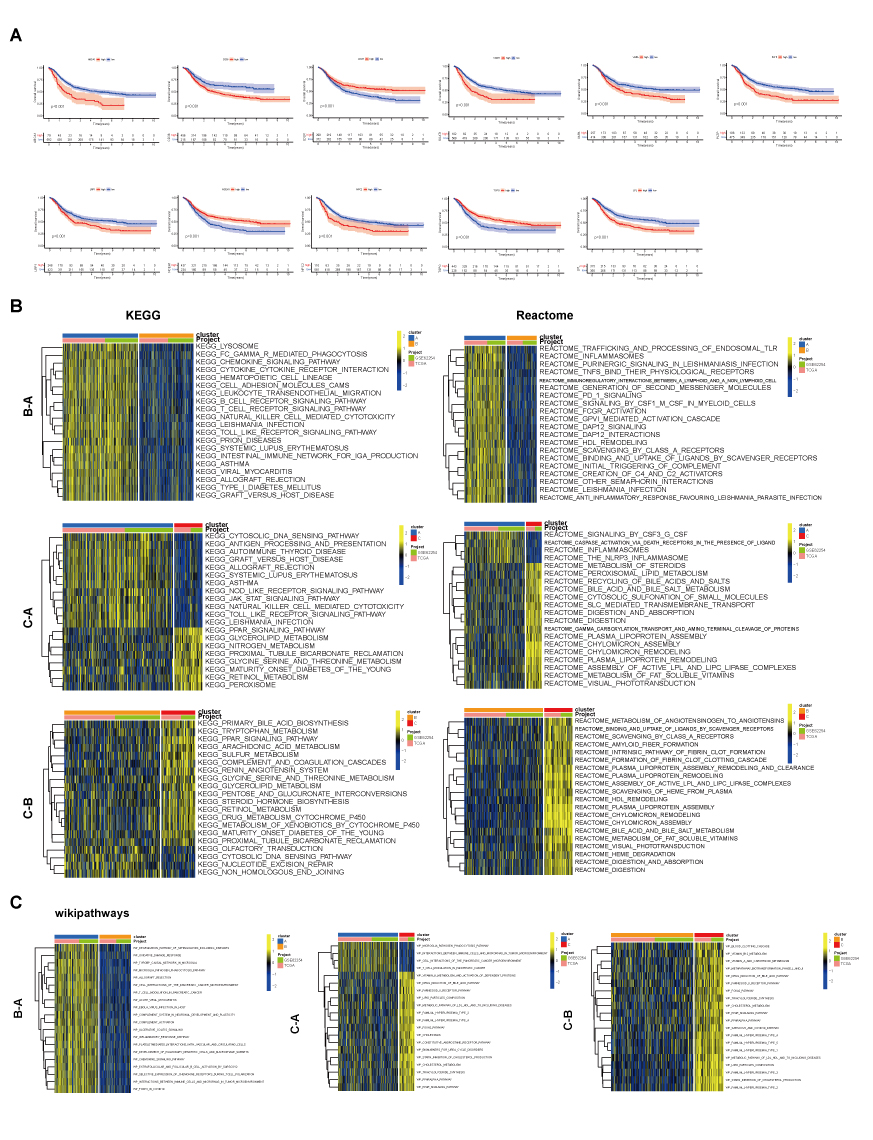

Supplement: Supplementary file 1 [file Image1.jpeg]

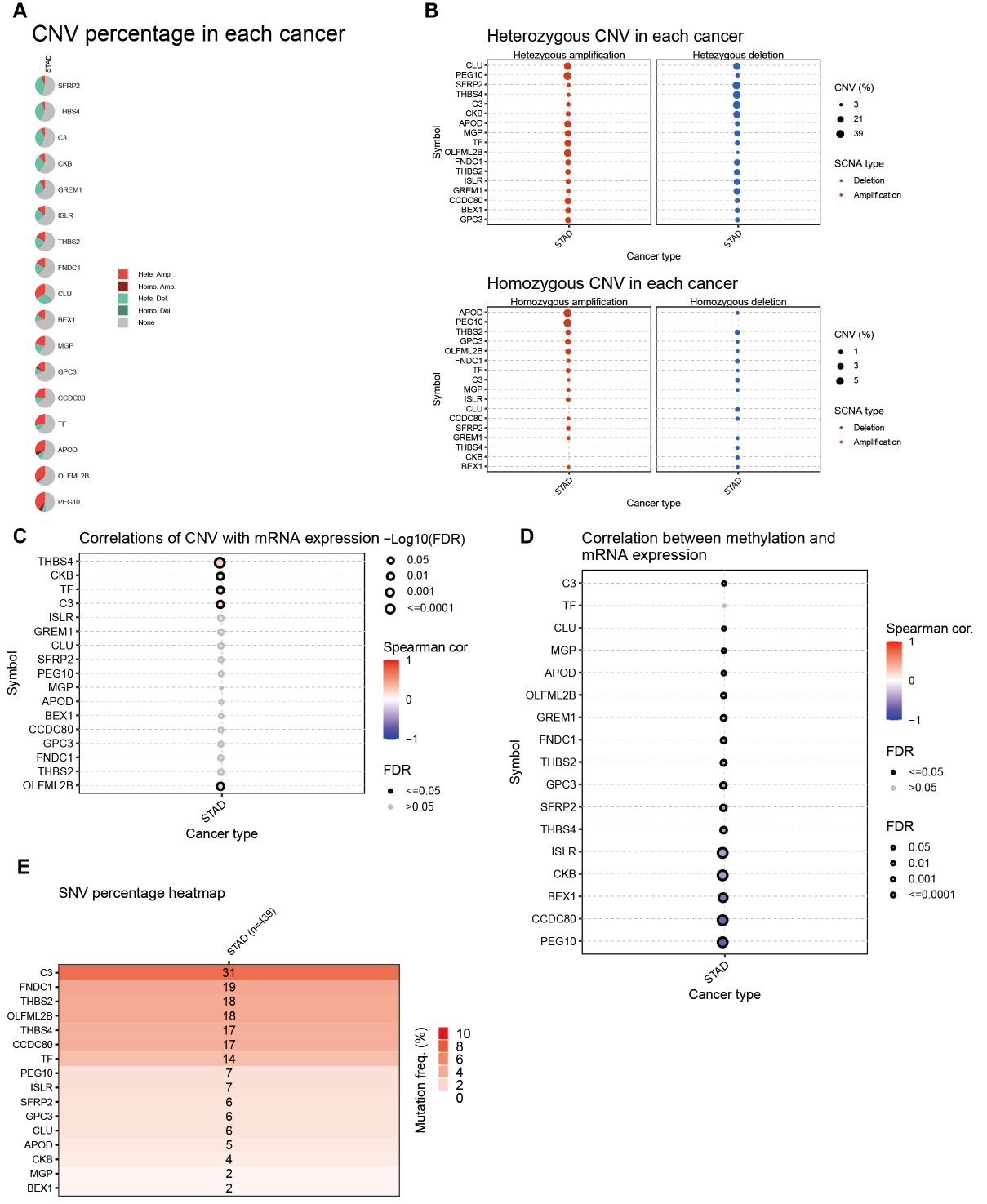

Supplement: Supplementary file 2 [file Image2.tif]

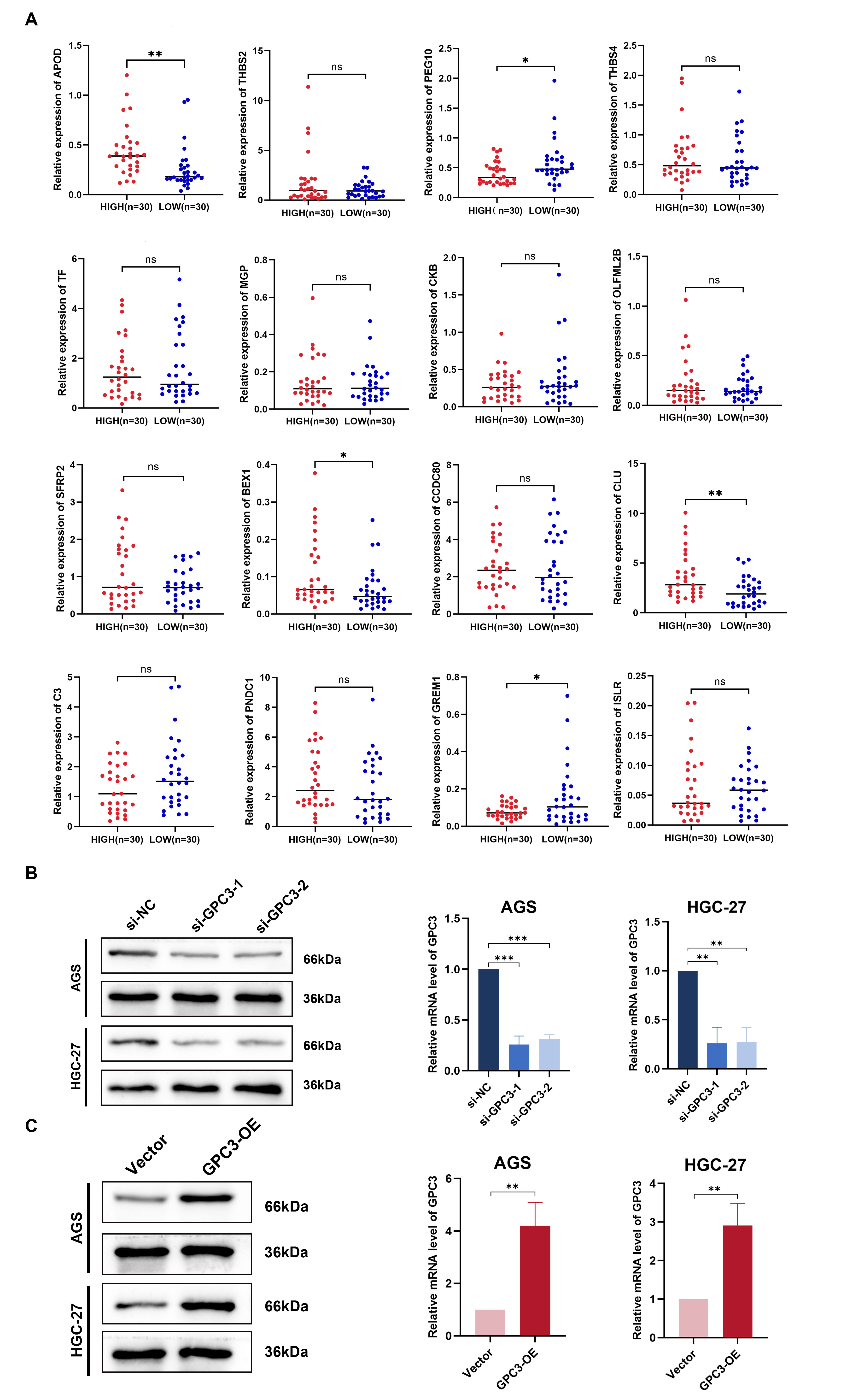

Supplement: Supplementary file 3 [file Image3.jpeg]

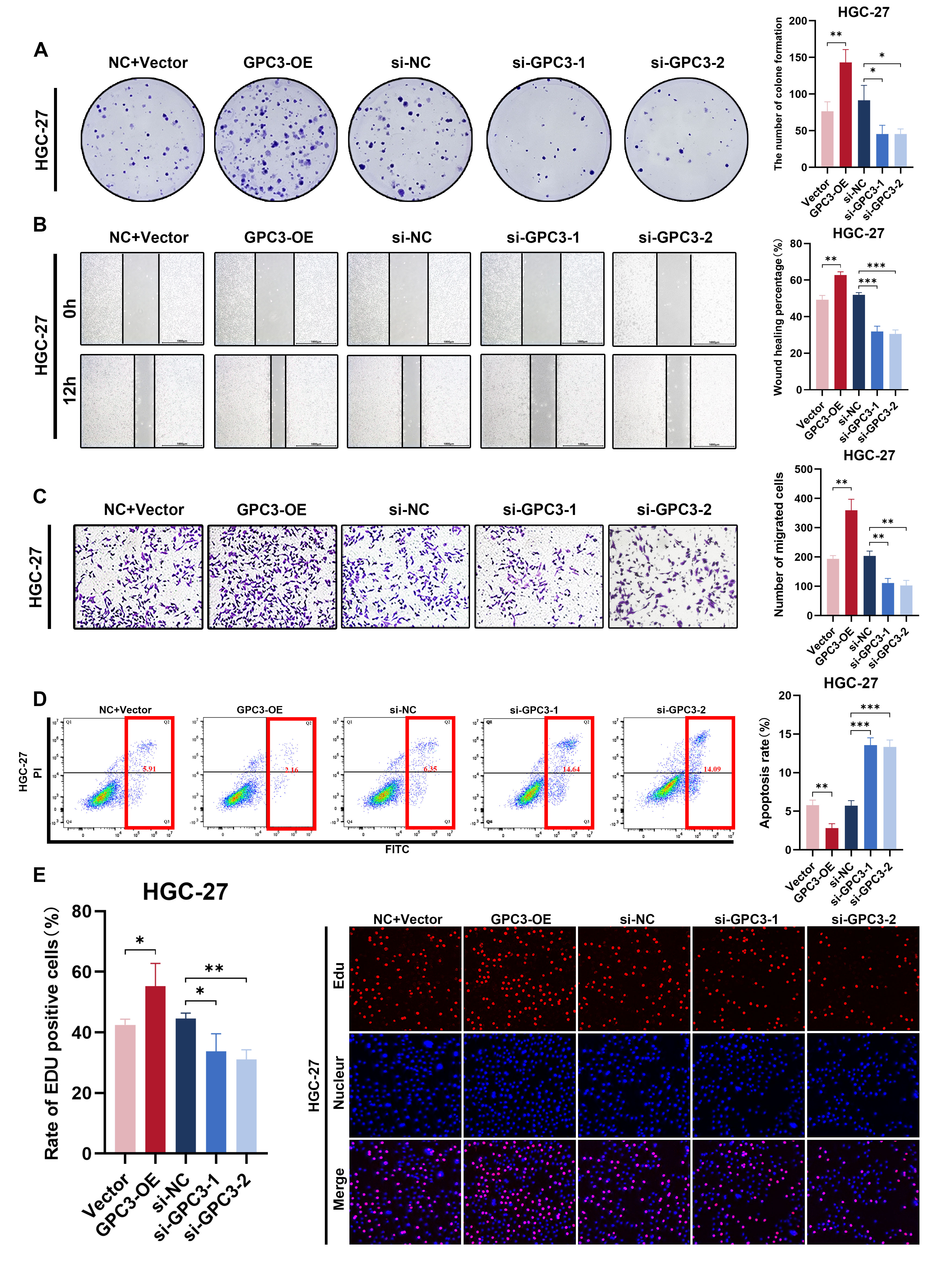

Supplement: Supplementary file 4 [file Image4.jpeg]
